# Effectiveness of SMS messaging for diarrhoea measurement: a factorial cross-over randomised controlled trial

**DOI:** 10.1186/s12874-020-01062-3

**Published:** 2020-06-30

**Authors:** Ryan Rego, Samuel Watson, Philbert Ishengoma, Philemon Langat, Hezekiah Pireh Otieno, Richard Lilford

**Affiliations:** 1 Warwick International Centre for Applied Health Research and Delivery, Warwick Medical School, University of Warwick, Coventry, UK; 2 Institute for Applied Health Research, University of Birmingham, Birmingham, UK; 3 The United Nations Human Settlement Program, Mwanza, Tanzania; 4 The United Nations Human Settlement Program, Nairobi, Kenya; 5 Brooklyn Economic Consulting, Nairobi, Kenya

**Keywords:** Diarrhoea, Surveillance, mHealth

## Abstract

**Background:**

Text messaging systems are used to collect data on symptom prevalence. Using a text messaging system, we evaluated the effects of question load, question frequency, and financial incentive on response rates and reported infant diarrhoea rates in an infant diarrhoea survey.

**Methods:**

We performed a factorial cross-over randomised controlled trial of an SMS surveying system for infant diarrhoea surveillance with treatments: financial incentive (yes/no), question load (1-question/3-question), and questioning frequency (daily/fortnightly). Participants progressed through all treatment combinations over eight two-week rounds. Data were analysed using multivariable logistic regressions to determine the impacts of the treatments on the response rates and reported diarrhoea rates. Attitudes were explored through qualitative interviews.

**Results:**

For the 141 participants, the mean response rate was 47%. In terms of percentage point differences (ppd), daily questioning was associated with a lower response rate than fortnightly (− 1·2[95%CI:-4·9,2·5]); high (3-question) question loads were associated with a lower response rate than low (1-question) question loads (− 7·0[95%CI:− 10·8,-3·1]); and financial incentivisation was associated with a higher response rate than no financial incentivisation (6·4[95%CI:2·6,10·2]).

The mean two-week diarrhoea rate was 36·4%. Daily questioning was associated with a higher reported diarrhoea rate than fortnightly (29·9[95%CI:22·8,36·9]); with little evidence for impact by incentivisation or question load.

**Conclusions:**

Close to half of all participants responded to the SMS survey. Daily questioning evoked a statistically higher rate of reported diarrhoea, while financial incentivisation and low (1-question) question loads evoked higher response rates than no incentive and high (3-question) question loads respectively.

**Trial Registration:**

The protocol was prospectively registered on ISRCTN on the 20th of March 2019 under number ISRCTN11410773.

## Background

Data from infectious disease surveillance systems are used in numerous ways, including the evaluation of public health interventions, providing early warning of outbreaks, and allowing for the proper allocation of resources. For children in low and middle income countries (LMICs), diarrhoeal disease is a key surveillance target, as it is globally the second highest cause of under-five mortality, as well as a large contributor to stunting and perhaps cognitive delay [1, 2]. There are two basic methods of disease surveillance: passive surveillance, such as the WHO’s Early Warning, Alert and Response System (EWARS), based on reports of targeted diseases from health facilities; and active surveillance, where households are visited randomly to ascertain diarrhoea rates. Both methods have strengths and weaknesses. Passive surveillance is inexpensive but underestimates diarrhoea rates (as not all affected patients visit reporting health facilities) (Rego R, Watson S, Lilford R: Systematic review of Diarrhoea measurement methods for under fives in LMICs, forthcoming). On the other hand, active surveillance, which is based on door to door questioning, may detect a higher proportion of cases but is costly and time-consuming (Rego R, Watson S, Lilford R: Systematic review of Diarrhoea measurement methods for under fives in LMICs, forthcoming).

Ninty percent of people in LMICs have access to basic mobile phones which can be used for health promotion and disease surveillance [3, 4]. In Southern Africa, for example, mobile phones have been used effectively for over a decade to ensure adherence to antiretroviral medication for HIV [5]. Mobile phones have also been used in Ghana during a demographic and health survey, and during the 2014–2015 West African Ebola outbreak [6, 7]. During the 2014–2015 Ebola outbreak in Liberia, mobile phone surveys were deployed to find Ebola cases, measure Ebola mortality, and evaluate care-seeking behaviours of Ebola patients [7]. The Liberian study, utilising a random list of phone numbers, conducted both phone call and and text message surveys. The study received a response rate of 15% to text messages and 13% to phone calls [7]. The results also showed a significant drop in response rates for both messages and phone calls between the first and second rounds of data collection – from 22 to 11% for text messages and 18 to 10% for phone calls [7]. L’Engle and colleagues (2018) used mobile phone voice message surveys to measure demographics and health behaviour in a Ghanaian non-emergency setting, obtaining a response rate of 31% [6]. The study also found that younger, urban, highly educated, and male respondents were more likely to respond to the mobile phone survey than face to face surveys – possibly resulting in bias [6].

It is clear that mobile phones can be used for disease surveillance. However, response rate in past studies was not very high and was influenced by numerous factors. Some factors may relate to the design of the system. We therefore decided to investigate the effect of certain factors of design on response rates. Given the importance of childhood diarrhoea, we selected this as the disease of interest for our study. Surveillance of childhood diarrhoea is also subject to a considerable amount of error given factors such as recall period and measurement frequency, with a majority of studies choosing either 24-h recall or 14-day recall (Rego R, Watson S, Lilford R: Systematic review of Diarrhoea measurement methods for under fives in LMICs, forthcoming). We therefore decided to measure the effects financial incentivisation (yes/no), recall period/questioning frequency (24-h vs 14-day), and question load (1-question/3-question) on response rates and reported diarrhoea rates to an SMS survey.

## Methods

This trial is reported in line with the Consolidated Standards of Reporting Trials (CONSORT) Statement [8]. The CONSORT Checklist can be found in Additional file 1.

### Study design

We conducted a factorial, multiple crossover randomised control trial (RCT) of three SMS messaging data collection formats: daily vs fortnightly messaging, incentivisation of 1000TZS (~ 0·40USD) per response vs no incentivisation, and a high question load (1-question) vs a low question load (3-question) survey instrument. This resulted in eight possible combinations of formats. The participants progressed through all eight treatment combinations in a random order over eight two-week rounds, between April and September 2019.

The study took place in three informal settlements in Mwanza, Tanzania, where participants were recruited with the assistance of local community leaders. Community leaders assembled adults who cared for at least one child between 6 and 60 months, had access to their own mobile phone, and expressed interest in participating. Two or three meetings were held in each of the three communities where potential participants were invited to attend at a time of their convenience. The meetings provided potential participants with an opportunity to learn more about the study, ask questions, and discuss the project with the study team and their peers. Those who wished to participate provided written consent by local field workers and were enrolled in the study. Consenting participants then completed a short demographic questionnaire.

### Randomisation

Eight study arms were formed such that at any time point one arm would be receiving one of the eight treatment combinations, with no arm receiving the same treatment combination at any time point. Treatment sequences were randomly generated by SW for each study arm with the restriction that no arm would receive the same incentive for more than two consecutive rounds or the same recall period for more than one round (Table 1). Further, each arm was sequenced to receive each of the eight treatment combinations over the study. RR then randomised participants at a 1:1:1:1:1:1:1:1 ratio into each arm using Microsoft Excel’s RAND function. Participants were blinded to their arm allocations and sequence.

**Table 1:** Treatment combinations for each arm (A-H) during each study round with treatments

		Round 1			Round 2			Round 3			Round 4			Round 5			Round 6			Round 7			Round 8		
		F	I	Q	F	I	Q	F	I	Q	F	I	Q	F	I	Q	F	I	Q	F	I	Q	F	I	Q
Arm	A	F	N	H	D	I	L	F	I	H	D	N	L	F	N	L	D	I	H	F	I	L	D	N	H
	B	D	I	L	F	N	L	D	N	H	F	I	H	D	I	H	F	N	H	D	N	L	F	I	L
	C	D	N	L	F	I	L	D	I	L	F	N	H	D	N	H	F	I	H	D	I	H	F	N	L
	D	D	N	H	F	I	H	D	I	H	F	N	L	D	N	L	F	I	L	D	I	L	F	N	H
	E	F	I	H	D	N	L	F	N	H	D	I	H	F	I	L	D	N	H	F	N	L	D	I	L
	F	F	I	L	D	N	H	F	N	L	D	I	L	F	N	H	D	N	L	F	I	H	D	I	H
	G	F	N	L	D	I	H	F	I	L	D	N	H	F	I	H	D	I	L	F	N	H	D	N	L
	H	D	I	H	F	N	H	D	N	L	F	I	L	D	I	L	F	N	L	D	N	H	F	I	H

Legend: 1) frequency (F), varying as daily (D) and fortnightly (F); 2) incentive (I), varying as incentive present (I) and no incentive (N); and 3) question load (Q), varying as high/3-question (H) and low/1-question (L)

### Procedures

**Fig. 1:**
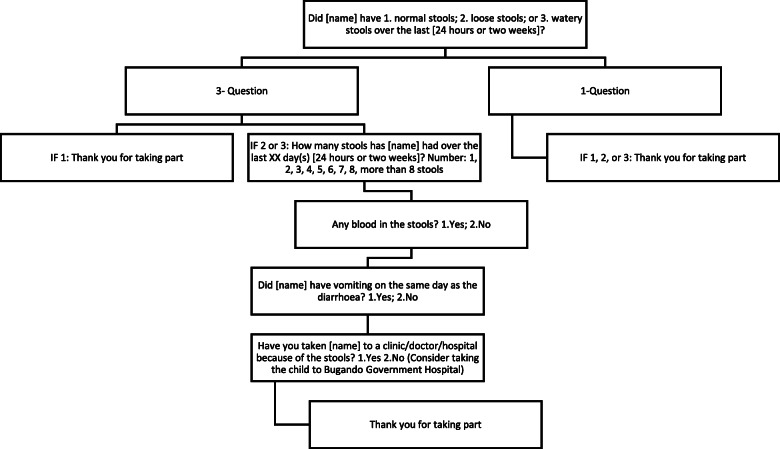
Questions in the text message survey

A text message, formatted according to the randomisation schedule, was sent via SMS message to participants between 10 AM and 11 AM on days due. On days due, all participants also received TZS 500 (~ 0·20USD) in airtime to cover the cost of responding to the survey. Participants receiving the incentive airtime payments were informed that this would be provided upon completion of all survey questions. If participants did not respond or complete the survey, they would receive two reminders – one after 4 h, and the second after a further 4 h. Responses were not accepted beyond 12 h from the initial message. Participants choosing to participate were sent the applicable survey as per their assigned arm (Table 1). Participants receiving the 1-question survey were asked if their child had normal stool, loose stools, or watery stools over the past 24 h or 14 days (dependent on frequency treatment) (Fig. 1). Participants receiving the 3-question survey were asked additional questions regarding blood in stool, vomiting, and health facility visits, if they reported loose or watery stool in the initial question (Fig. 1).

We conducted nine qualitative interviews to determine barriers and motivators to the SMS surveys. Participants for qualitative interviews were chosen purposively to select those who had answered with different levels of frequency and to represent each study settlement. Sampling ensured that in each settlement, one person who never answered, one person who answered consistently, and one person who answered with varying consitency was interviewed. The interviews took place in KiSwahili and were conducted using a semi-structured guide exploring questions on daily life, attitudes towards the SMS system, and how the SMS system fits into their daily life (Additional file 2). The interviews were transcribed and later translated into English for analysis.

### Outcomes

The first outcome of the study was the complete survey response rate. Complete response was pre-defined as completing all questions in > 70% of the daily surveys sent in a two-week round, or completing all questions in the fortnightly survey. The second outcome was the reported rate of having diarrhoea during the two-week round, measured as having any number of loose or watery stools in the past 2 weeks. The third outcome was the attitudes towards the different surveying strategies, as uncovered in the qualitative work.

### Sample size

We conducted a simulation-based analysis of the design. We calculated delta, the minimal detectable treatment effect, for power of 80% and type I error rate of 5%. We assumed that the interaction effect sizes were half the size of the direct effect of each treatment. A baseline response rate of 50% was assumed as this was the most conservative value in terms of power. We also assumed an intra-class correlation coefficient of 0·05 for the proportion of variance at the individual level. Under these assumptions, the minimum detectable average treatment effect was 7·5 percentage points.

### Statistical analysis

Complete response was analysed using a standard model for factorial trials [9]. A multivariable logistic regression model was estimated, containing indicators for each treatment and all treatment interactions, as well as demographics (participant sex, education, age, and household income) and random effects at the individual level. Dummy variables to adjust for time and area were also included. Average marginal treatment effects for each treatment in absolute terms (percentage point difference) were then estimated from each model.

The second outcome, diarrhoea rate, was examined in an identical way for complete responses – again with a logistic regression model, using the reported presence of loose or watery stool at any point during the two-week period as the dependent variable. Qualitative interviews were examined through the use of a codebook. This enabled us to identify themes and how often they were elicited in the semi-structured interviews described above. Themes of paticular attention included time, convenience, comfort, and social harms. The codebook was then examined to determine the most frequent themes.

All data were monitored daily for any issues with receipt of the data. Due to the failure of the mobile phone network, rounds two and seven were repeated at the end of the survey period (disregarding any data from the first attempt of rounds two and seven).

## Results

### Participant demographics

**Fig. 2:**
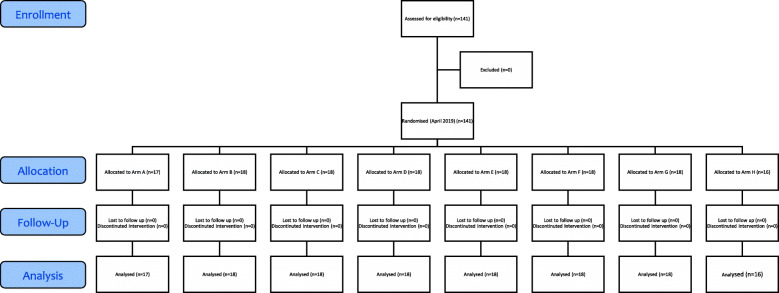
CONSORT Flow Chart

In April 2019, one-hundred and forty-one respondents were recruited and randomised into one of eight arms. Figure 2 presents the CONSORT flow chart. There were no withdrawals, and all respondents who wished to take part were eligible. Table 2 reports the summary statistics for the study cohort. The average age of the respondents was 28·9 years – with most (92·2%) having completed primary school or above. Respondents were predominantly female (97·9%), and most (51·1%) had a household income below 50,000TZS (21·75USD) a month. The average household size was 5·5 people.

**Table 2:** Summary statistics of demographics and quantitative study outcomes for all participants, by study arm

		A	B	C	D	E	F	G	H	ALL
**Total, n (%)**		17 (12·1)	18 (12·8)	18 (12·8)	18 (12·8)	18 (12·8)	18 (12·8)	18 (12·8)	16 (11·3)	141
**Age,** Mean (SD)		28·9 (6·2)	28·4 (6·8)	27·6 (5·2)	30·4 (9·2)	30·3 (6·7)	29·3 (6·7)	29·9 (8·1)	27·9 (7·2)	29·1 (7·0)
**Education,** n (%)	None	0	0	0	1 (5·6)	0	0	2 (11·1)	1 (6·3)	4 (2·8)
	Some Primary	0	0	0	1 (5·6)	1 (5·6)	1 (5·6)	3 (16·7)	1 (6·3)	7 (5·0)
	Finished Primary	10 (58·9)	10 (55·6)	13 (72·2)	11 (61·1)	9 (50·0)	12 (66·6)	8 (44·4)	11 (68·8)	84 (59·6)
	Some Secondary	1 (5·9)	4 (22·2)	0	2 (11·1)	0	4 (22·2)	3 (16·7)	2 (12·5)	16 (11·3)
	Finished Secondary	6 (35·3)	4 (22·2)	3 (16·7)	2 (11·1)	8 (44·4)	1 (5·6)	2 (11·1)	0	26 (18·4)
	Some Tertiary	0	0	2 (11·1)	0	0	0	0	1 (6·3)	3 (2·1)
	Finished Tertiary	0	0	0	1 (5·6)	0	0	0	0	1 (0·7)
**Sex,** n (%)	Male	0	0	0	1 (5·6)	2 (11·1)	0	0	0	3 (2·1)
	Female	17 (100%)	18 (100%)	18 (100%)	17 (94·4)	16 (88·9)	18 (100%)	18 (100%)	16 (100%)	138 (97·9)
**Income,** n (%)	> 50,000	8 (47·1)	10 (55·6)	8 (44·4)	12	8 (44·4)	9	8 (44·4)	9 (56·3)	72 (51·1)
	50,000-100,000	5 (29·4)	4 (22·2)	7 (38·9)	6 (35·3)	7 (38·9)	4 (22·2)	4 (22·2)	7 (43·8)	44 (31·2)
	100,000-500,000	2 (11·8)	3 (16·7)	2 (11·1)	0	3 (16·7)	3 (16·7)	4 (22·2)	0	17 (12·1)
	500,000+	0	0	0	0	0	1 (5·6)	1 (5·6)	0	2 (1·4)
	Decline to say	2 (11·8)	1 (5·6)	1 (5·6)	0	0	1 (5·6)	1 (5·6)	0	6 (4·3)
**Area,** n (%)	Unguja	6 (35·3)	6 (35·3)	6 (35·3)	6 (35·3)	6 (35·3)	6 (35·3)	7 (38·9)	7 (43·8)	50 (35·5)
	Igogo	6 (35·3)	7 (38·9)	7 (38·9)	6 (35·3)	6 (35·3)	6 (35·3)	6 (35·3)	5 (31·25)	49 (34·8)
	Kilmahaewa	5 (29·4)	5 (27·8)	5 (27·8)	6 (35·3)	6 (35·3)	6 (35·3)	5 (27·8)	4 (25·9)	42 (29·8)
**Household Size,** Mean (SD)		5·6 (2·2)	4·9 (1·7)	5·7 (2·2)	5·4 (3·2)	5·1 (1·6)	5·9 (2·6)	6·8 (3·0)	4·8 (2·1)	5·53 (2·4)
**14-day Diarrhoea Prevalence,** %		34·67	27·12	38·26	42·86	40·62	30·36	24·00	52·38	36·35
**Complete Response Rate,** %		55·15	42·75	79·86	48·61	44·44	38·89	34·72	32·81	47·33

### Survey response rates

Over the course of the study, between April and September 2019, 8215 surveys were distributed: 7655 daily texts and 560 fortnightly texts, with an even split between the high question load (3-question) and low question load (1-question) surveys, and incentive and no incentive. These can be broken down into 1122 child-rounds of observation (each round lasting 2 weeks). The trial concluded in September 2019 when all arms progressed through all treatment combinations.

**Fig. 3:**
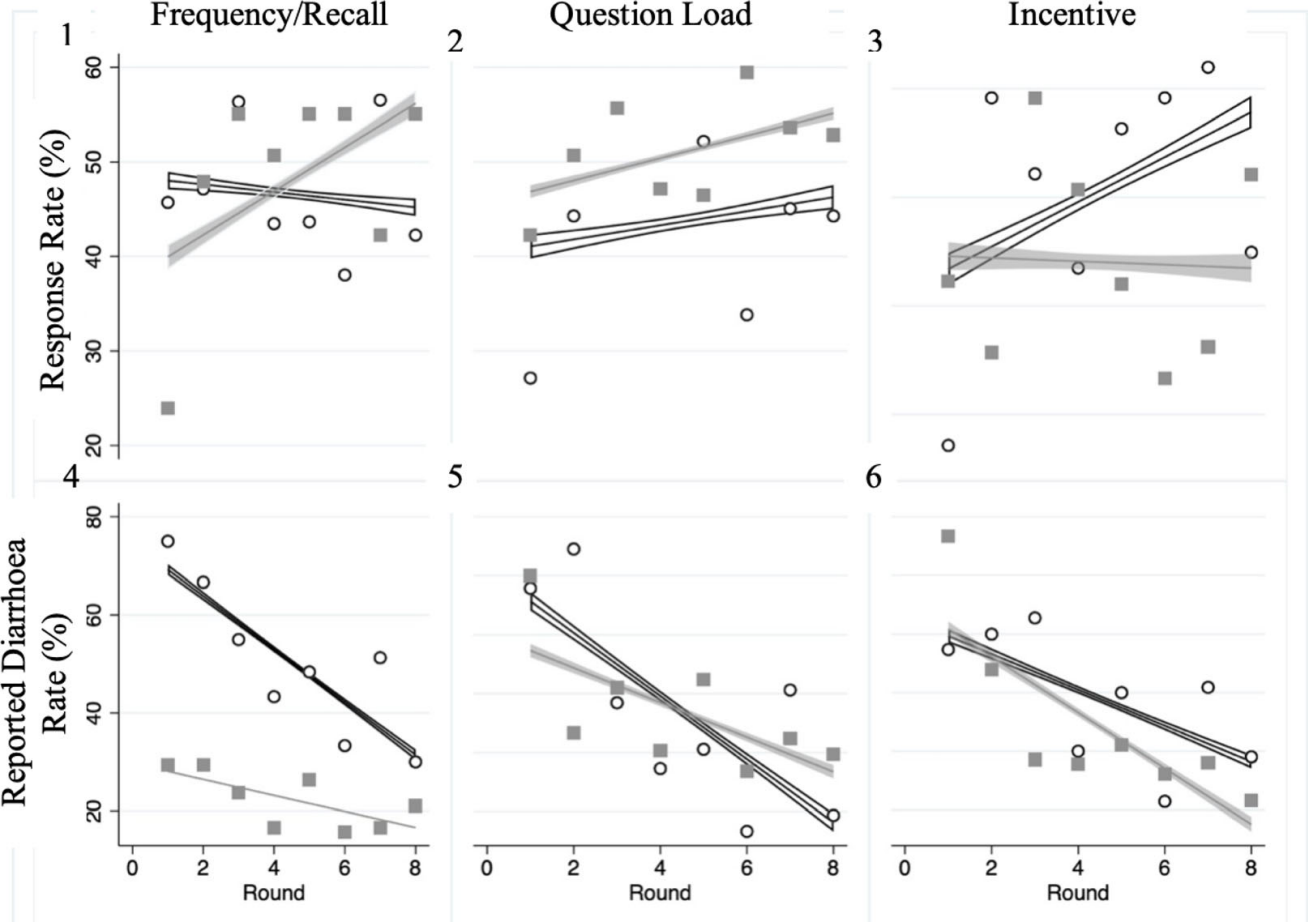
Mean response rates (1–3) and estimated diarrhoea rates (4–6) over all eight rounds to the SMS Survey, broken down by treatment. Legend: 1 and 4: daily (circle points) Vs Fortnightly (square points) surveys; 2 and 5: 3-Question (circle points) Vs 1-Question (square points) surveys; and 4 and 6: Incentive (circle points) Vs No Incentive (square points). Trend lines are displayed with corresponding 95%CI

**Fig. 4:**
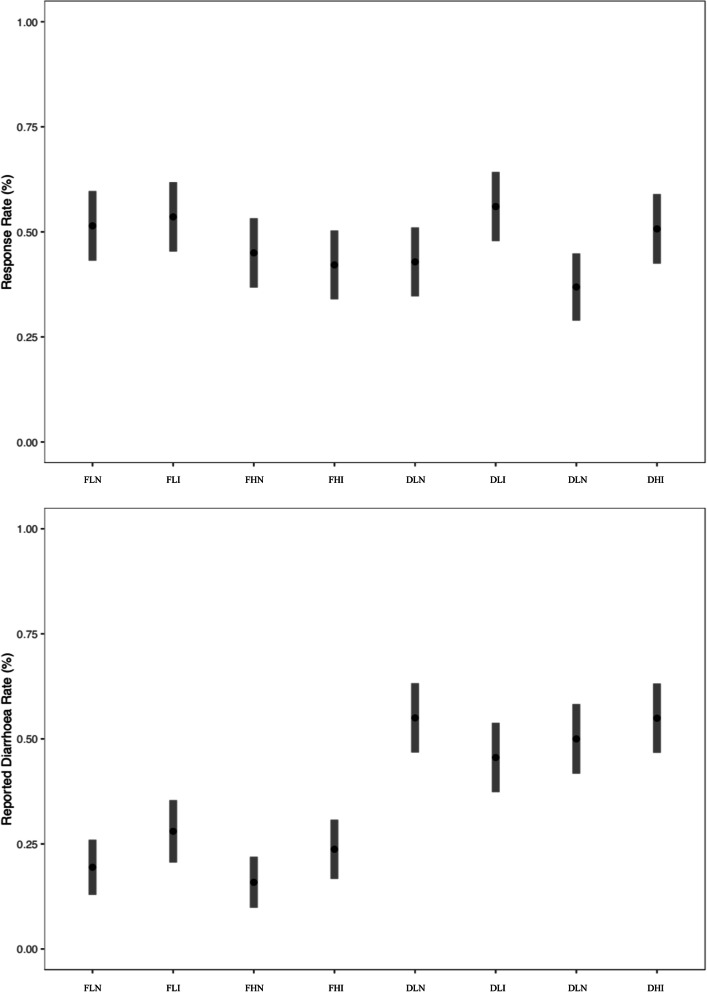
Mean response rate (above) and estimated diarrhoea rate (below), and 95%CIs, of treatment combinations. Legend: F: Fortnightly Questioning; D: Daily Questioning; L: Low Question Load (1-Question Survey); H: High Question Load (3-Question Survey); N: No Incentive; I: Incentivisation

The mean response rate was 47%. Daily questioning had a similar mean response rate to fortnightly questioning (46·6% vs 48·0%); the 3-question survey was lower than the 1-question (43·8% vs 51·0%); and the incentivsed surveys was higher than the surveys without incentive (50·6% vs 44·0%) (Fig. 3). When examining mean response rates by interactions between treatments, there was little evidence of any interaction between treatments, other than response rates being lower when daily questioning and the 3-question survey were combined (Fig. 4). Response rates increased as the study progressed (Fig. 3).

Table 3 reports the results from the adjusted model-based analysis. Daily questioning was associated with a non-significant reduction in the response rate by a 1·2 percentage point difference (ppd) (95%CI[− 4·9,2·5]), compared to fortnightly questioning. The 3-question survey was associated with a significant reduction of response rates by 7·0ppd (95%CI[− 10·8,-3·1]) compared to the 1-question survey. Incentivisation was associated with a significant increase in response rates by 6·5ppd (95%CI[2·6,10·2]) compared to no incentive.

**Table 3:** Estimated Adjusted Treatment Effects and Effects of Demographic Factors on Response Rate

	Adjusted Treatment Effect (percentage point difference, (95%CI))
Daily Recall vs 14 day Recall	-1·2 (− 4·9,2·5)
3-question Survey vs 1-question Survey	−7·0 (− 10·8,−3·1)
Incentive vs No Incentive	6·4 (2·6,10·2)
Age of Respondent (continuous in years)	1·1 (0·2,2·1)
Beyond Primary Education vs Primary Education or Lower	11·7 (−1·6,25·1)
Low Income (below 50,000TZS) vs Middle or High Income	−3·8 (−16·2,8·6)
Study Round (continuous)	0·9 (0·0,1·7)
Kilimahewa vs Igogo	-3·4 (−18·6,11·2)
Unguja vs Igogo	−6·0 (−21·2,9·1)

There was also evidence that respondent age affected response rates, with each additional year of age being associated with an increased in response rate by 1·1ppd (95%CI[0·2, 2·1]), as did time, with a 0·9ppd (95%CI[0·0,1·7]) increase per round (Table 3). Having education beyond the primary stage was associated with an increase in response rates by 11·7ppd (95%CI[− 1·6,25·1]) when compared to having primary education or lower. Having a low income (below 50,000TZS) was associated with a decrease in response rate by 3·8ppd (95%CI[− 16·2,8·6]) when compared to middle or high income (Table 3).

### Estimated Diarrhoea rates

Overall, 36·4% of the 14-day child-rounds reported diarrhoea. When broken down by treatment, daily questioning had an estimated diarrhoea rate of 51·2% (compared to 21·9% for fortnightly questioning); the 3-question survey had a 36·3% estimated diarrhoea rate (compared to 36·4% for the 1-question survey); and the incentivised surveys had a 38·7% estimated diarrhoea rate (compared to 33·6% for surveys without incentivisation) (Fig. 3). When looking at the impact of interactions between interventions on diarrhoea rate, we see a similar trend, with all treatment combinations that included the fortnightly survey having a similar lower estimated rate, regardless of interaction (Fig. 4). The estimated diarrhoea rate appeared to decrease as the study progressed (Fig. 3).

Table 4 reports the results from the model-based analysis. Compared to fortnightly questioning, daily questioning was associated with a significant increase in the estimated diarrhoea rate, with an adjusted treatment effect of 29·9ppd (95%CI[22·8,36·9]). There was no evidence to suggest that the 3-question survey had a significant impact on the estimated diarrhoea rate, with an adjusted treatment effect of 0·0ppd (95%CI[− 6·0,5·9]). There was little evidence indicating that financial incentivisation had a significant impact on the estimated diarrhoea rate, with the incentive raising the estimated diarrhoea rate by 3·0ppd (95%CI[− 3·1,9·0]).

**Table 4:** Estimated Adjusted Treatment Effects and Effects of Demographic Factors on Estimated Diarrhoea Rate

	Adjusted Treatment Effect (percentage point difference, (95%CI))
Daily Recall vs 14-day Recall	29·9 (22·8,36·9)
3-question Survey vs 1-question Survey	−0·0 (− 6·0,5·9)
Incentive vs No Incentive	3·0 (− 3·1,9·0)
Age of Respondent (continuous in years)	-1·2 (−2·2,-0·2)
Beyond Primary Education vs Primary Education or lower	3·7 (−10·2,17·6)
Low Income vs Middle or High Income	-0·3 (−12·9,12·3)
Study Round (continuous)	−2·9 (−4·3,−1·5)
Kilimahewa vs Igogo	4·5 (−10·5,19·6)
Unguja vs Igogo	-1·6 (−16·7,13·6)

Evidence showed an impact by respondent age, with each additional year in age associated with a decrease in the estimated diarrhoea rate by 1·2ppd (95%CI[− 2·2,− 0·2]), but not by other demographics (Table 3). Evidence also indicated a decrease in the estimated diarrhoea rate over the course of the study by 2·9 ppd. per round (95%CI[− 4·3,− 1·5]) .

### Qualitative findings

A high degree of acceptance with the SMS surveying system was observed during the analysis of the qualitative interviews. Participants were accustomed to using mobile phones, as they use them in daily life for work, communicating with friends and family, and studying. Participants reported that the messages were not perceived as intrusive and that late morning receipt of messages was convenient. Participants further reported appreciation of the reminders, as they were sometimes busy when the first message came. Participants did, however, prefer infrequent questioning, stating that they believed that they were able to recall diarrhoea over 14-days.

Participants generally stated that while the incentive was appreciated, it did not factor into their decision whether or not to take part in the survey. Participants reported appreciation of being able to feedback on their child’s health and that the messages encouraged the carers to pay more attention to their child’s health.

Participants were mixed regarding preference towards face to face surveys vs SMS surveys, but for the most part, appreciated the ease and privacy of SMS surveying. Participants suggested that in the future SMS and face to face be integrated, with more emphasis on education rather than purely surveying.

## Discussion

We conducted an individual level factorial multiple crossover randomised control trial in Mwanza, Tanzania to estimate the effects of questioning frequency, question load, and incentivisation on response rates to an SMS survey on under-five diarrhoea in urban informal settlements. The study also included analyses of the effects of demographics on the response rate; the effects of questioning frequency, question load, and incentivisation on the reported diarrhoea rate; and a qualitative examination of attiudes towards text message surveys. The principle findings of the study are that SMS messaging can be a suitable means of disease surveillance in LMICs, with response rates of around 50% in our study, but that results can be impacted by the methodologies used: financial incentivisation is associated with an increase in the response rate, increased questioning loads is associated with a decrease in the response rate, and frequent questioning with short recall periods is associated with a decrease in the reported diarrhoea rate.

### The impact of treatments on the response rate

The complete response rate over all eight rounds was 47% - a proportion higher than reported in previous similar studies: 15% in Liberia during the Ebola Outbreak, and 31% in Ghana during a demographic and health survey [6, 7]. Reasons for the higher response rate include differences between the study sites, health topic, and study recruitment (with our study recruiting consenting participants, whereas the aforementioned studies randomly messaged unconsulted participants). Evidence from our study additionally indicates that daily questioning (24-h recall) had a similar response rate to the fortnightly survey (14-day recall), suggesting that after 14 days of daily questioning respondent fatigue did not set in, as has been suggested in past studies [10]. Further supporting that fatigue did not set in, there was increase in the response rate over time.

There did appear to be a lower response rate for the 3-question survey (when compared to the 1-question survey) – suggesting that the 3-question survey was burdensome for the respondents. This is in line with past studies, including Bhavnani and colleagues (2014), who suggested that fatigue might occur if participation required a high amount of effort [10]. This is supported by our finding that when daily questioning is combined with the 3-question survey, there is an additional lowering in response rate. This reduction in response rate may have been even more apparent if regardless of response participants were given all three questions, rather than only being given all three questions if the participant reported diarrhoea.

The incentive did yield a statistically significant increase in the mean response rate. While inconsistent with the qualitative findings that incentivisation did not factor into participants’ decisions whether or not to take part in the survey, this is consistent Hopkins and Gullickson’s (1992) meta-analysis on the impact of financial incentivisation on survey response, which found that financial incentivisation increased response rate by 19% when given with the survey (prior to completion) and by 7% when given after the survey. The latter figure is similar to the 6·4ppd increase in response rate observed in our survey through provision of an incentive after survey completion [11].

### The impact of demographics on the response rate

Those with higher education were more likely to respond, with those who had progressed beyond the primary stage of education responding at a rate 11·7ppd higher than those with primary education or below. This finding was also seen in L’Engle and colleagues (2018) study on demographic and health surveys Ghana [6]. L’Engle and colleagues surveyed a nationally representative sample using an 18 question demographic and health survey to determine response rates to a mobile phone survey [6]. This study used pre-recorded voice messages in which participants would respond by inputting a certain number on their dial pad [6]. Comparing the results of the mobile phone survey to two similar nationwide surveys which used face to face surveying, the study estimated that populations with no education answered the mobile phone survey at a rate 5 to 18 ppd. less than a face to face survey [6]. The study also estimated that populations with secondary education or above answered the mobile phone survey at a rate 27 to 29 ppd. higher than a face to face survey [6]. L’Engle and colleagues conclude that while mobile phone surveys are a promising tool for data collection, differential response rates by varying demographics could introduce bias if adjustments were not made.

### The impact of treatments on the reported Diarrhoea rate

Diarrhoea was reported in 36% of complete child-rounds – yielding an incidence of 9 episodes per child year. While this number is slightly higher than previously reported in urban East Africa, the finding can be explained on the basis that all participants in the previous studies were presented a 14-day recall period [12]. When restricting the analysis to the 14-day recall period, we estimated an incidence of 6 episodes per child year, in line with previous studies. This is considerably lower than the estimated incidence of 13 episodes per child-year for 24-h recall. The higher diarrhoea rate estimated for the daily survey with 24-h recall, when compared to the fortnightly survey with 14-day recall, provides support of recall bias, whereby respondents forget events that occur over long periods [13, 14]. Feiken and colleagues (2010) report prevalence dropping from 18% for 24–48 h recall to around 5% in 11–13 day recall [13, 14]. Zafar and colleagues (2010) report that severe diarrhoea is twice as likely to be reported as moderate diarrhoea during longer recall periods [13, 14].

Incentive and survey type did not influence reported diarrhoea rates. Of interest, however, reported diarrhoea rates did decrease markedly over subsequent rounds. We hypothesize three (non-exclusive) reasons for this. First, the survey may have created a heightened awareness of diarrhoea risk and child health (as reported in the qualitative work), resulting in better WASH practices; second, respondents may have been embarrassed by constantly reporting diarrhoea [15]; third, respondents may have telescoped answers at the beginning of the survey (recalling from a longer period than the stated recall period).

### Strengths and weaknesses

There are two substantial weaknesses in this study. 1) As the questioning frequency treatment included variation of both frequency and recall period – with fortnightly questioning asking about the past fortnight, and daily questioning asking about the past day, it is not possible to determine if the differences associated with this particular treatment were due to the frequency or the period of recall. 2) The study was unable to ascertain the impact of perception bias and if participants truly understand what defines a case of diarrhoea. For example, in a previous study, Voskuijl and colleagues (2017) found that parents of infants with severe acute malnutrition in a Malawian hospital were only able to identify 75% of loose or watery stools as such (loose or watery stools being identified by observation by a health care provider) [16].

This study has several strengths. The study took place in an urban East African city with a fairly representative culture and geography of other urban East African areas, so we believe that the results are generalisable to similar settings. Further, the study data provides results which are not only consistent throughout the study, but also build of past literature. Bhavnani and colleagues (2014) discussed the possibility of respondent fatigue through frequent, in depth, questioning which we provide evidence for [10]. Hopkins and Gullickson (1992) found evidence that incentivisation is associated with increased response rates, which we also find evidence for, but in the novel form of an SMS survey [11]. Similarly, Feiken and colleagues (2010) and Zafar and colleagues (2010) found evidence for recall bias during in-person surveys for diarrhoea, which we also see in our novel SMS survey [13, 14]. Finally, L’Engle and colleagues (2018) found a substantial association of demographics, such as education, on response rate in their SMS survey, but, due to their use of uninformed participants, had a low response rate [6]. Our use of informed partipants resulted in a higher response rate.

## Conclusion

SMS surveying is a feasible method of collecting data on child health among populations with high levels of access to mobile phones. There are several variations in the system which may affect response and reported diarrhoea rates. Financial incentivisation (compared to no financial incentivisation) increases the response rate but does not impact the reported diarrhoea rate. A high question load (3-questions compared to 1-question) decreases response rate, particularly when done so at a high frequency, but does not impact the reported diarrhoea rate. Daily questioning and recall (compared to 14-day questioning and recall) does not impact response rate, but dramatically increases the reported diarrhoea rate.

When conducting standard in-person active surveillance, our results call for the need to standardise the methodologies used to minimise undesirable variation in results. These standardised methodologies should use incentivisation and low question loads to maximise response rate, while using a short recall period to minimise recall bias.

Future research is needed in this field, however, including evaluation of SMS surveillance systems in other populations, such as those in rural areas; evaluation of questioning frequency and recall period separately; and further evaluation into the ability of parents to correctly identify diarrhoea.

## Supplementary information

**Additional file 1.**

**Additional file 2.**

## Data Availability

The datasets used and/or analysed during the current study are available from the corresponding author on reasonable request.

## Abbreviations

BSREC: Biomedical and Scientific Research Ethics Committee
CI: Confidence Interval
CONSORT: Consolidated Standards of Reporting Trials
EWARS: Early Warning, Alert, and Response System
HIV: Human Immunodeficiency Virus
ISRCTN: International Standard Randomised Controlled Trial Number
LMICs: Low and Middle Income Countries
NIHR: National Institute for Health Research
PPD: Percentage Points Differences
RCT: Randomised Control Trial
SMS: Short Message Service
TZS: Tanzanian Shillings
USD: United States Dollars
WASH: Water, Sanitation, and Hygiene
WHO: World Health Organization
