# Supplementary material for: Effectiveness of SMS messaging for diarrhoea measurement: a factorial cross-over randomised controlled trial
Source: BMC Med Res Methodol. 2020 Jun 30;20:174. doi: 10.1186/s12874-020-01062-3 (PMC7325153; doi:10.1186/s12874-020-01062-3)
Supplement: Supplementary file 2 — Additional file 2. [file 12874_2020_1062_MOESM2_ESM.docx]

**Introduction:** Thank you for your interest in taking part in this interview. My name is Philemon Langat, and this is [NAME]. We work with UN-Habitat on the project using SMS messages to measure diarrhoea in Mwanza, which you may recall recently agreeing to be a part of. This project has been funded and is run by the University of Warwick in the UK. As part of the project, we hoped to interview 10 parents involved in the study about specific aspects of the diarrhoea survey, including for example, what worked well, what didn’t work so well, what you found difficult or problematic etc. We want to speak to a range of people. You were selected to be one of the parents that we would (initially) like to interview. We will ask you a few questions – it will up to 30 minutes. You’ll see me and my colleague [NAME] write down notes on what you say in order for us to write about the interview later. We will not record any information which could be used to identify you, and we will not audio or video record this interview. Our discussion will either be reported in a summary, or with short quotes, ensuring that there is no identifying information. You will be able to find more information on this on the participant information sheet which I have handed you, and on your signed consent form. Are you happy for us to begin?

| **Questions** | **Probes** |
| --- | --- |
| Section 1: Daily Life, Overall Impressions and Use  First, I’d like to find out a little more about you and your family. Could you walk me through a typical day in your life, and a day in your household?  We’ll get into a little more detail on this, but how did the SMS phone system fit into your daily life, and what were your overall impressions? | - What time do you wake up? - Do you work? What do you do? - Who do you live with? - Who cares for the children? - Who has what responsibilities in your home? - Who do you interact with on a daily basis? - How often do you use your mobile? And for what? - When do you go to sleep? - Were they convenient? - Were they annoying? - Did you have to alter your life at all? - Did they come at a convenient time? - Was it good that you got reminders? - Did you change any behaviours based on this?   - Perhaps hygiene behaviours; or paying more attention to child health/stools |
| Section 2: Incentive and Airtime  You may have noticed that while we always sent you some airtime before the survey, TZS500, to make sure that you have enough airtime to complete the survey; we also occasionally sent you airtime after the survey, TZS1000, to say thank you. Did knowing that you would receive this airtime after the survey influence if you responded or not, and how you responded? Why? | - Did the incentive make it worthwhile to take part or not? - Did you feel pressured or not to give answers you think we’d want if we gave you incentive? - Did you talk to your friends or not about this at all? If so, what did you discuss? - Was TZS1000 a substantial incentive, or a small one? - Did caring or not about the incentive change over time? |
| Section 3: Questioning Frequency  You may have also noticed that we adjusted how often we texted you: sometimes texting you every day, asking about diarrhoea over the past 24 hours; and sometimes texting you once in two weeks, asking about diarrhoea in the past two weeks. Did you prefer one of these methods? And Why? | - How was the frequency of the questioning – was it the right amount, or should it be more or less? - Did you feel that the questions were comfortable to answer, or where they too intrusive into your life? - When we asked you about what happened two weeks ago, rather than in the past day, were you able to remember? - Was receiving airtime a factor in you preferring frequent or infrequent messages? |
| Section 4: Question Levels  As part of the survey, we sometimes asked a few more questions to parents reporting diarrhoea in their children – questions on blood in stool, vomiting, frequency of diarrhoea, and hospital visits. At other times, we did not. Did you have a preference to us asking extra questions or not? | - When we asked you several questions, did you find this to be intrusive? Or did you prefer to be able to share more with us? - Did we ever tell you to go to the hospital? If so, did you appreciate us doing this, and did you go? |
| Section 5: Methods of Improvement  We’re always looking for ways to improve our work, and greatly value your feedback. Would you be able to tell me a few of the things that you liked about the survey; things which made it more likely for you to take the survey; and things which you think we should introduce in the future?  How about things that you did or did not like about the survey; things which made it easy or difficult for you; and things you think we should change?  Would you be interested or not in taking part in a similar study in the future; and would you or would you not recommend a study like this to your friends? | - Did you feel comfortable answering the survey? Would you rather talk to a person, or do you prefer the privacy by phone? - Did your friends, family, and community pressure you to either complete or not complete the survey? - Was money a factor in completing or not completing the survey? - Were you able to use the technology easily, or was it confusing? - Could you easily understand the questions? |
| Conclusion  Is there anything else which you could like to share with me? |  |

Thank you for taking part in this interview. We greatly appreciate your feedback, and will be using it in our future work. You’ll continue to receive our text messages until August. Feel free to reach out to us if you have any questions. Remember that you can contact us on the information on the participant information sheet that I have given you.
